# Bridging the gap in healthcare: creating an online health information prototype for individuals with intellectual disabilities to promote equity and knowledge transfer

**DOI:** 10.3389/fpubh.2023.1194892

**Published:** 2023-09-12

**Authors:** Lisa T. Dam, Petra Heidler, Isabel King

**Affiliations:** ^1^International Cooperative Cross-Border Interdisciplinary Doctoral Programme in Educational and Communication Sciences, University of Applied Sciences Burgenland, Eisenstadt, Austria; ^2^Institute International Trade and Sustainable Economy, IMC University of Applied Sciences Krems, Krems an der Donau, Austria; ^3^Department of Health Sciences, St. Pölten University of Applied Sciences, St. Pölten, Austria; ^4^Department of Exercise Physiology, School of Health, University of the Sunshine Coast, Sunshine Coast, QLD, Australia

**Keywords:** knowledge transfer, health information, intellectual disabilities, equity, website prototype, easy-to-read language, Austria

## Abstract

**Introduction:**

The prevalence of intellectual disabilities (ID) in Austria is estimated to be approximately 1% of the population. Growing awareness of the major challenges faced by individuals with ID, including difficulties in comprehending written health information, communication barriers with healthcare professionals, and limited inclusion in health education research, necessitates concerted efforts to address these issues. The utilization of guidelines promoting easy-to-read language, web accessibility, and usability can significantly enhance access to health information and improve health literacy among people with ID. The “LUCHS-Gesundheitsinformationen (Health Information)” project aimed to improve knowledge transfer of health information for people ID by creating a website prototype.

**Methods:**

Unstructured interviews were conducted with two focus groups (*n* = 20) people with ID and their caretakers to elicit relevant topics and ensure the quality, comprehensibility, and usability of a website prototype. A convenience sample of 20 adults employed in sheltered workshops of the Caritas Lower Austria West facility who met the inclusion criteria participated.

**Results:**

The prototype addressed four main topics, namely nutrition, medical specialization, first aid, and patient information, offering comprehensive information using easy-to-read language and pictorial content. The website prototype followed web accessibility suggestions and incorporated external resources, such as brochures and emergency contact details, to enhance usability and provide a reliable source of health information for individuals with ID.

**Discussion:**

The findings suggest that a website format is a feasible means of providing accessible online health information for people with ID. However, further research is warranted to explore the potential of digital health applications for individuals with ID, as inclusion in the Austrian register for digital health applications necessitates meeting multiple quality standards, thereby instilling trust among the target group.

## Introduction

According to Austrian reports (1), in 2003, approximately one out of a hundred individuals had an intellectual disability (ID). Intellectually disabled people suffer from two major obstacles when accessing information about their health. First, they face a significant barrier concerning the comprehension of written information in any media since they are mostly provided in high complexity (1, 2). The second issue is the knowledge transfer between health professionals and patients with intellectual disabilities. Such barriers can lead to misunderstandings in communication and may result in poorer health in patients (3).

Considering the greater vulnerability of people with ID to health disparities due toco-existing impairments combined with limited intellectual functioning and adaptive behaviors, the transfer of health knowledge is gaining importance, as well as addressing the obstacles of people with ID. People with ID often suffer from co-existing impairments since they have a higher chance of developing physical disabilities, hearing or vision impairments, mental health problems and communication disorders (4). Although the awareness of accessibility issues rises, people with ID are underrepresented in health education and research and suffer from insufficient patient empowerment (5).

Using digital devices, such as laptops and mobile devices and technologies like mobile applications and online resources, in combination with their increasing availability, can significantly improve the health outcomes of people with ID and the general population. Digital literacy is supported and iteratively revised by monitoring access, utilization and impact of health-related knowledge transfer (6, 7). Furthermore, guidelines regarding digital content, such as the Web Content Accessibility Guidelines as well as ones referring to information in general as “Easy to Read” (“Leicht Lesen”), help improve the accessibility of provided information (2). Another method to simplify the process of information comprehension is the visual research method “Photovoice” (5, 8). It equips participants with cameras to help record issues and reflect upon them, thus enabling patients to communicate their health concerns or discuss them with peers. Photovoice is commonly used in education, community development and public health research since it effectively makes knowledge transfer more inclusive (5, 8, 9). However, the existing literature on communication barriers for people with ID mostly focuses on missing tools or concepts and the negative impact of these shortcomings, or rather seeks the views and experiences of people without learning disabilities, such as professionals and paid carers. Therefore, focusing on detecting solutions to improve knowledge transfer regarding health information for people with ID, we considered the perspective of this particular population in the development of the LUCHS website prototype to cover health-related information as possible means of knowledge transfer. This paper draws upon the lessons learned from this case study in developing the health literacy strategy in the area (10). These lessons are transferrable and can provide insight into effective and collaborative working in community-based health communication and health promotion.

## Context

Participants of this project are people with mild to moderate intellectual disabilities (ID), which can be categorized as 6A00.0 and 6A00.1 according to ICD-11 or F70 and F71 in the previous ICD-10 (11, 12). The WHO describes disorders of intellectual development as “*a group of etiologically diverse conditions originating during the developmental period characterized by significantly below average intellectual functioning and adaptive behavior”* (12). 6A00.0 (ICD-11) describes “*mild disorder of intellectual development*” and was previously classified as F70. On the other hand, 6A00.1 or the former F71 details *“moderate disorder of intellectual development.”* Mild or moderate disorder of intellectual development can be distinguished by “*significantly below average intellectual functioning and adaptive behaviour*” (11, 12). Common experiences are of people with ID are difficulties regarding understanding texts. Often, they are able to learn skills necessary for living independently with consistent appropriate support (12).

Easy-to-read language applies various guidelines and suggestions for writing texts targeting people with ID. Common guidelines in German-speaking countries are “Leichte Sprache” (German: easy language) and “Leicht Lesen” (German: easy reading), and in English-speaking countries is “easy-to-read language” (13, 14). “Leicht Lesen” offers guidelines for writing texts and serves as a quality seal for German texts written in easy-to-read language (15). Dam et al. (10) focus on the guideline of Inclusion Europe, which consists of the most important rules as also found in other corresponding guidelines (16). Some of the main rules consist of using simple words, short sentences, and examples to explain, writing numbers as digits and using sans-serif fonts to increase the comprehensibility of texts for people with ID (16–18).

Since 1999, the Web Accessibility Initiative has published guidelines to improve Internet access for people with and without disabilities (19, 20). Suggestions include providing textual alternatives for non-text content to enable adaptation to users’ diverse needs, such as translations into easy-to-read language and increasing readability for screen readers. Furthermore, website creators should provide multiple ways leading to specific content to simplify website navigation. Such suggestions for improved accessibility apply to people with and without disabilities, as difficult navigation will reduce the usage of websites. For example, Krug (21) emphasizes the benefits of persistent navigation of websites, particularly home buttons, search bars, and “breadcrumbs.” Breadcrumb navigation, or “breadcrumbs,” provides an easy way to keep track of the current location within a website. As usability also contains accessibility, Krug (21) refers to different guidelines for web accessibility. For example, next to short suggestions concerning alternative texts, Krug advises designing the website in a way that is operable with the keyboard to make it accessible for people unable to utilize a mouse.

## Materials and methods

A literature review was performed regarding accessibility, usability and easy-to-read guidelines, and the inclusion of two health professionals. Furthermore, the expertise of two health professionals provided valuable insights and knowledge concerning dietetics and occupational therapy. A qualitative research approach was followed as the heterogeneous target group comprises people with ID. Unstructured interviews with two single focus groups of people with ID and two caretakers were conducted to elicit relevant topics and ensure quality concerning the comprehensibility of the written information and usability of the website prototype. Health researchers established the use of focus groups in social action research and preventive health education. Through a moderated conversation, the technique provides the researcher with insight into the participants’ complicated personal experiences, beliefs, perceptions, and attitudes. Through a moderated conversation, the technique provides the researcher with insight into the participants’ complicated personal experiences, beliefs, perceptions, and attitudes (21). Each focus group consisted of 10 adults working in sheltered workshops classified as 6A00.0 and 6A00.1 (mild to moderate intellectual development disorder) according to ICD-11. Participants were recruited from the Caritas Lower Austria West facility. The facility managers approved to conduct the interviews with their employees, and all participants gave their consent during a personal meeting and with the support of their caretakers. As inclusion criteria the above-mentioned ICD-11 diagnosis as well as interest in participating and health related topics must be fulfilled. Other potential participants not meeting these criteria have been excluded. Data collection took 3 months in total. The first interviews took place with both focus groups to identify their main interests in health-related topics. The main task of the first focus group was to provide feedback on the written and pictorial content focusing on comprehensibility. They performed quality assurance on printed versions of the texts accompanied by the respective pictures and photographs. Their caretakers provided notes on the focus group’s feedback while the participants read the content the first time and after reading the revised versions to enable the researchers to gather more in-depth comments, as the interviews concerning their feedback took place 2 to 3 weeks after providing them with the content. The period of 3 weeks ensured that all participants could read the texts before asking the focus group for feedback. After implementing their feedback, a website prototype was created.

The first focus group provided quality assurance regarding understandability, whereas the second focused on the usability of the website prototype. The only exception concerning content feedback was video.

The resulting website prototype contains four main topics acquired with the focus groups: nutrition, medical specialization, patient information and first aid. Each topic offers a broad range of information in easy-to-read language supported by pictorial content ranging from healthy recipes to information about health professions, chest compressions and the Austrian electronic health record.

As stated in an earlier work by Dam et al. (10), the Lower Austrians Federal Government collaborates with multiple non-profit organizations operating sheltered workshops and assisted living according to the Employment of People with Disabilities Act (Disability Employment Act). The Austrian disability law is based on the Disability Employment Act, the Federal Disability Act and the Federal Disability Equality Act. One partner mentioned above is Caritas Lower Austria West, which currently operates 23 sheltered workshops, commercial shops, and recycling workshops employing people with ID. Non-probability methodologies (e.g., convenience sampling, quota sampling, and snowball sampling) address people by providing detailed inputs to answer the questions under investigation (22, 23).

## Results

After identifying four main health-related topics with the support of both focus groups, the authors created a website prototype. The four topics examined by the website prototype were nutrition, medical specialization, patient information, and first aid. Each topic offers a broad range of information in easy-to-read language supported by pictorial content ranging from healthy recipes to information about health professions, chest compressions and the Austrian electronic health record. The authors adapted an open-source website theme by wordpress.com following the main suggestions of web accessibility.

The nutrition section provided general information about the food pyramid with detailed texts about the different food groups such as fruits, vegetables, grain, dairy products, meat, sweets, and junk food. Adjusted to the participants’ interests, the website prototype included information such as yo-yo dieting, overweight and healthy dieting, and healthy recipes. A dietician performed proofreading the information in this section.

The medical specialization section described the main tasks of various medical specialists and the procedures of main examinations and treatments. Furthermore, the texts included information about disease prevention and tips for self-care in that matter. The website focused on oculists, gynecologists, ear-nose-throat (ENT) physicians, dermatologists, and orthopedists.

First aid concentrated on treating unconscious or unresponsive patients and burn victims. The website contained instructions for chest compressions, mouth-to-mouth ventilation, and recovery positions. In addition, the website prototype explained the different stages of burns, their distinctive features, and immediate measures. This section is supposed to be a reference text for both focus groups, as multiple participants stated that they completed first aid courses. Moreover, the content included the Austrian emergency numbers, including 122 and their area of expertise and listed the main information the call-taker needs to help most efficiently.

The fourth section, patient information, contained information about health professionals such as dieticians and occupational therapists. Similar to the medical specialization section, the content provided information about examinations and treatments performed by the respective health professionals. Furthermore, as the media discussed the Austrian electronic health records (ELGA) and piqued the participants’ interest, this section briefly describes ELGA. Another feature of this section was a link to a brochure for patients with disabilities about doctor-patient consultation by the Lower Austrian Government (24).

**Figure 1:**
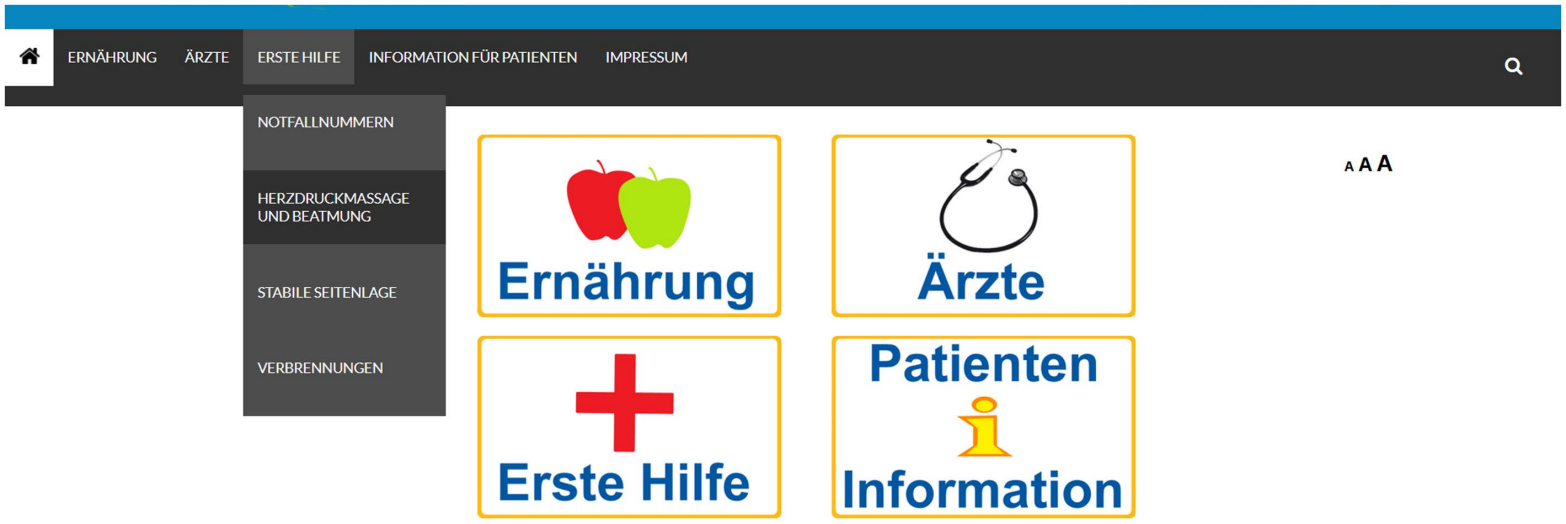
Landing page of prototype.

Figure 1 displays the landing page of the prototype used in this study. The focus was on a visually reduced design from wordpress.com, which was adapted by the authors for this research. It focused on easy webpage navigation and navigation symbols familiar to the participants such as the home button, the search symbol as well as the triple A for changing font sizes. The buttons for the four topics of the prototype consist of the written names in addition to commonly used graphics or symbols in Austria such as apples for nutrition or a red cross for first aid.

## Discussion

Health is a broad topic, even if reduced to four main topics per the participants’ interests. The creation of the content showed challenges in reducing written text lengths while still providing the crucial information necessary for comprehension and increasing health literacy. As mentioned by Inclusion Europe et al. (16), reduce information displayed on the screen to help avoid scrolling. This proved challenging as adherence to this suggestion would mean separating the text into smaller sub-chapters leading to more complex navigation. Even if following guidelines for usability by Krug (21, 25) explaining the importance of “easy navigation” and the implementation of multiple navigation ways, the authors found that a compromise between both advises might provide a more feasible realization of the website prototype.

This example shows the necessity of a compromise between content and usability as too much content or a too broad range of topics described in a website result in reduced usability since finding information becomes more difficult. While self-testing websites containing vast variety of health information targeting patients without ID, we found navigating websites with extensive content difficult as too specific information is convoluted. This is especially true if patients do not exactly know their search goals. Examples containing medically proofed information were the Austrian and German version of NetDoktor (26).

We decided on health topics based on the results of interviews with the focus groups. Photovoice as described by Jurkowski (5), Latz et al. (8), and Budig et al. (9) might be another feasible method to determine health-related topics of interest. Concerning the utilized web technology, further tests might help ascertain more suited web technologies to create websites targeting people with ID. Game-based learning, defined as a game with clearly set learning outcomes by Plass et al. (27) and Shaffer et al. (28), could be included in the website prototype as further development to enhance knowledge transfer. Game-based learning and providing information of the website prototype in other media, such as mobile applications for tablets or smartphones, would provide further insight regarding the effectiveness of knowledge transfer.

This study provides the basis for the research by Dam et al. (10), measuring knowledge transfer in health-related topics and creating a questionnaire prototype usable for people with ID. The findings will result in further recommendations and a feasible questionnaire for people with disabilities.

**Figure 2:**
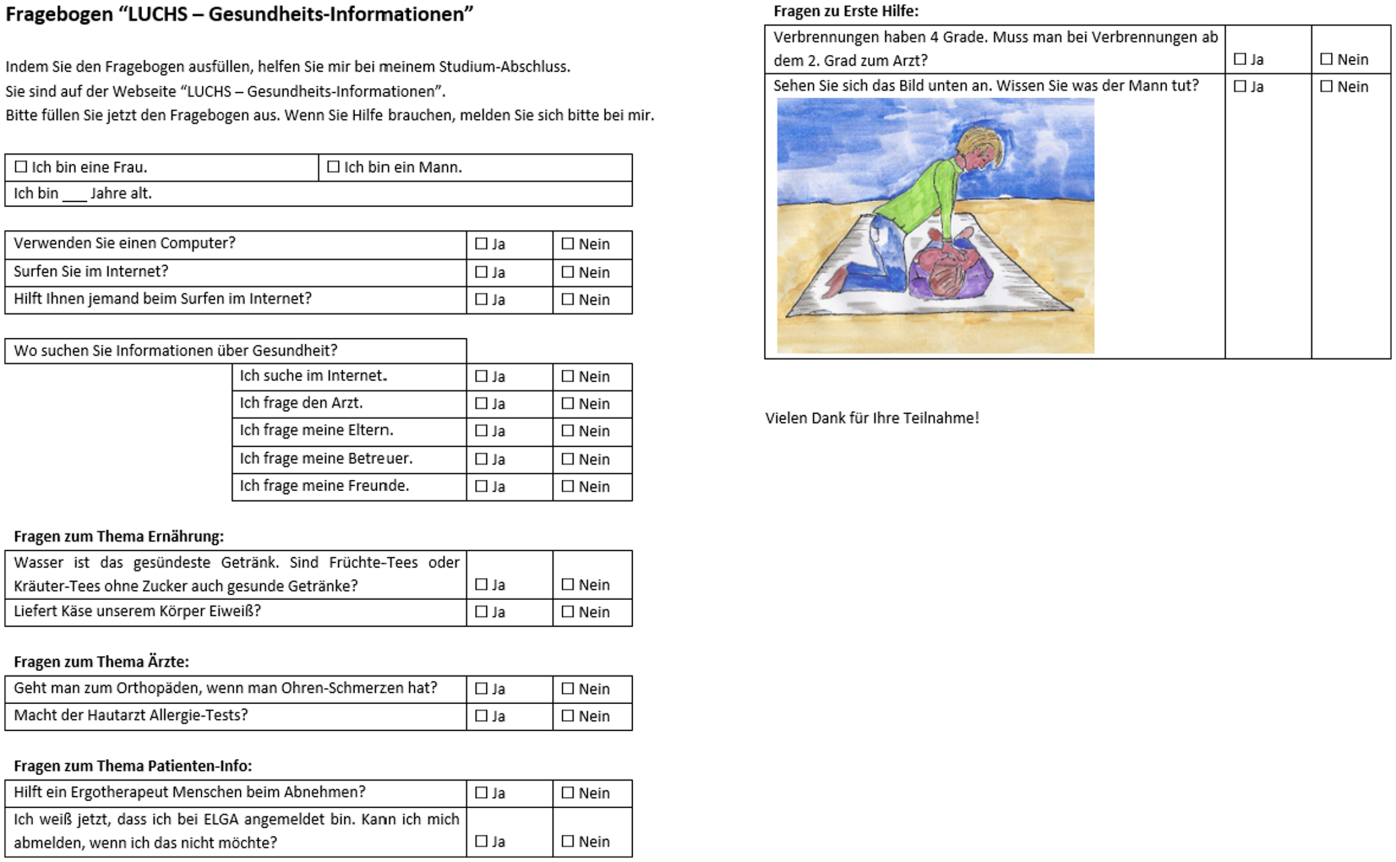
German questionnaire based on this research by Dam et al. (10).

Figure 2 illustrates the questionnaire developed based on the results of this research by Dam et al. (10). It contains a short introduction summarizing the authors verbal explanations, followed by a demographic part as well as questions about their computer usage and health information sources. The second part of the questionnaire consists of questions to measure knowledge transfer about nutrition, medical specialization, patient information, and first aid.

## Limitations

This study has several limitations that need to be acknowledged. Firstly, the study suffers from selection biases attributed to the vulnerability of the group and limited access to potential participants, resulting in a convenience sample and potential selection bias. Additionally, the narrow inclusion criteria used in the study has led to a small sample size, which limits the generalizability of the findings to the broader population. Moreover, the web-based self-administered survey method used in this study introduces the possibility of response bias among the participants. Furthermore, the lack of updated and representative data on intellectual disabilities in Austria poses a challenge in creating equitable online health information for this group. To address these limitations, further research with a more systematic and inclusive sampling approach is necessary to improve the representativeness and generalizability of the findings and support future developments in this area.

## Conclusion

In conclusion, a website is a feasible way to offer accessible health information. Provided, that the content creators follow guidelines for easy-to-read language (29–30) and web accessibility as well as usability guidelines (19–21).

We recommend further research concerning digital health applications focusing on people with ID. Digital health applications or digital therapeutics (German: Digitale Gesundheitsanwendungen “DiGA”) are defined as “*evidence-based therapeutic interventions for patients by means of qualified software programs to prevent, manage, or treat medical conditions*” (31, 32). The registration of digital applications requires multiple quality assurance and adherence to quality standards (33). We assume that required adherence to standards as well as examination of experts might increase the target group’s trust. The authors expect that with further research approved digital therapeutics can be adapted by other countries for their populations’ needs and other specific target groups.

## Data availability statement

The original contributions presented in the study are included in the article/supplementary material, further inquiries can be directed to the corresponding author.

## Ethics statement

The studies involving humans were approved by Ethics Committee of Lower Austria. The studies were conducted in accordance with the local legislation and institutional requirements. Written informed consent for participation in this study was provided by the participants.

## Author contributions

All authors listed have made a substantial, direct, and intellectual contribution to the work and approved it for publication.

## Conflict of interest

The authors declare that the research was conducted in the absence of any commercial or financial relationships that could be construed as a potential conflict of interest.

## Publisher’s note

All claims expressed in this article are solely those of the authors and do not necessarily represent those of their affiliated organizations, or those of the publisher, the editors and the reviewers. Any product that may be evaluated in this article, or claim that may be made by its manufacturer, is not guaranteed or endorsed by the publisher.
